# Do Sex and Gender Have Separate Identities?

**DOI:** 10.1007/s10508-024-02933-2

**Published:** 2024-08-06

**Authors:** Gonzalo R. Quintana, James G. Pfaus

**Affiliations:** 1 Departamento de Psicología y Filosofía, Facultad de Ciencias Sociales, Universidad de Tarapacá, Arica, Arica y Parinacota Chile; 2 Department of Psychology and Life Sciences, Charles University, Prague, 18200 Czech Republic; 3 Center for Sexual Health and Intervention, Czech National Institute of Mental Health, Klecany, Czech Republic

**Keywords:** Sex, Gender, Intersex, Binary, Spectrum, Sexual orientation

## Abstract

The largely binary nature of biological sex and its conflation with the socially constructed concept of gender has created much strife in the last few years. The notion of gender identity and its differences and similarities with sex have fostered much scientific and legal confusion and disagreement. Settling the debate can have significant repercussions for science, medicine, legislation, and people’s lives. The present review addresses this debate though different levels of analysis (i.e., genetic, anatomical, physiological, behavioral, and sociocultural), and their implications and interactions. We propose a rationale where both perspectives coexist, where diversity is the default, establishing a delimitation to the conflation between sex and gender, while acknowledging their interaction. Whereas sex in humans and other mammals is a biological reality that is largely binary and based on genes, chromosomes, anatomy, and physiology, gender is a sociocultural construct that is often, but not always, concordant with a person’ sex, and can span a multitude of expressions.

## Introduction

For over half a century, the scientific community has faced a growing controversy regarding the meaning of gender and its conflation with sex. When the meaning of things is at stake, society often relies on two sources to settle the dispute: the dictionary and/or the scientific literature. Depending on the language, the former often relies on systematic research performed by scholars who study the historical, sociological, economical, and geographic development and use of the language. They aim to describe, more than prescribe, the way language is used, its meaning, rules, etymology, derivations, pronunciation, etc. (Oxford, 2021a; RAE, 2021a). The latter relies in a logical, evidence-based efforts aiming to describe natural and social phenomena where new terms are coined and organized. Thus, each construct must follow a sound theory and rationale, respect the delimitations in its definition, and challenge them in light of new evidence, in order to systematically describe and organize the nature of reality (see Kuhn, 1962). Our understanding of sex and gender has expanded and deepened into more complex constructs. As a result, their definitions and intersections are not clear-cut and have become fertile territory for debate and confusion, especially when nonscientific terminology and a conflation of the two are used to advance a particular set of beliefs or sociopolitical agenda.

The present schism lies between two apparently irreconcilable perspectives. On the one hand, there are those who believe sex is a binary construct, biologically determined, immutable, observable, measurable; aiming to describe, among other nuances, how sexually differentiated animals and humans function and reproduce, and recognizing the existence of disorders related to it (e.g., Byrne, 2023; Hilton et al., 2021; McCarthy & Arnold, 2011). On the other hand, there are those who not only decry this as dogma, but also believe sex and gender are both socially constructed and exist on a spectrum that includes the binary distribution of male and female, but also a rainbow of different gender identities and expressions between or beyond male and female (e.g., Butler, 1999). The issue is scientific in nature, yet appears ontological, and is made even more complex when considering arguments from justice, power, inclusion, class, identity, intersectionality, and privilege into the mix (e.g., Hyde et al., 2019; Johnson, 2017; Raymond, 1994). The lofty goal of settling the debate would have significant consequences for science, medicine, legislation, and people’s lives. This is especially true given the already negative environment that sexual minorities face (e.g., Mountjoy et al., 2016), the recurrent conflation of sex and gender in research (e.g., Bhargava et al., 2021), the latest trends of science denial (e.g., Hansson, 2017), and the many social issues that are currently related to this matter. Therefore, it is imperative to find ways to clarify the discussion points, especially when the perspectives taken by “opposing sides” may not be as irreconcilable as they seem.

## The Schism Between Sex and Gender

The word “gender” dates from the fourteenth century. It is a word first used to describe a group, class, or type to which things belong, to some extend what “genre” is for the English language (RAE, 2021b). Its status as a synonym for a person’s sex became more common in the twentieth century (Oxford, 2021b). Gender as we know it now was derived from the concepts of “sex status” and “sex role,” referring to the defined patterns of what a person is, and the “behavioral counterpart of the ideal or expected position defined by a status,” respectively (Parsons, 1940; p. 43). Money (1955) is credited with introducing the concept of gender to describe the sense or subjective state of one’s masculinity or femininity. However, Bentley (1945) had actually published an article on the topic prior to Money, wherein he referred to gender as the socialized obverse of sex, with the qualifying terms being “feminine” and “masculine.”

The feminist movement went further, making a distinction between sex and gender, where the former described natural features related to one’s anatomy and reproductive function, and the latter was the result of socialization, a process where individuals were taught to behave in accordance with their gender and societal expectations of gender roles and stereotypes (Butler, 1999; de Beauvoir, 1949/1989; Stockard, 2006). This gendered socialization process taught women to behave in feminine ways (e.g., soft, nurturing, caring, submissive) that were perceived as less valuable than those masculine ways taught to men (e.g., strong, dominant, assertive, aggressive, etc.). Fighting for equal rights, women faced uphill social and legal battles against limitations that were based on their sex or womanhood (Cott, 1987). The idea of rejecting and breaking down traditional gender roles and stereotypes also led to a reevaluation of the binary relation between femininity and masculinity. A culture was formed around gender, challenging the devaluation of the feminine, while also shifting ideas from an emphasis on the question of women’s sameness or difference from men toward deconstructing the concept of “woman” in the wide diversity of individuals it represents (Pelak et al., 1999; Taylor & Rupp, 1993). Suddenly, the concept of womanhood, and therefore female gender, was being redefined outside the binary conceptions of sex, and some even rejected its value as a construct altogether (Poirot, 2009; Radicalesbians, 1970). More recently, scholars began to understand gender as an intrinsic part of individual’s identity, but also as a construct that cannot be fully captured through the binary conception of man or woman, masculine or feminine, male or female, which also varies throughout history, ethnicities, cultures, social classes, etc. (e.g., Butler, 1999). The notion of gender as something more than the binary became popular, giving space for other identities such as non-binary, gender queer, and agendered (see Richards et al., 2016). The discussion has become even more complex when considering transgender individuals, who experience a discrepancy between their biological sex or “sex assigned at birth” and their gender expression and identity (e.g., Coleman et al., 2012). This has become controversial in sports disciplines that use sex as a determining factor to allow individuals to compete in a male or female category. The inclusion of transgender individuals has fostered much dispute about fairness and safety (e.g., BBC, 2021; IRFU, 2022; Joel & Fine, 2022). Indeed, the only alternative to the two categories are the Paralympic games, where trans-persons, people with disorders of sex development (DSDs), or non-binary individuals qualify to participate.

## Conflation of Sex and Gender

From a biologically deterministic perspective, sex serves reproduction, and sexual reproduction requires a merging of DNA carried by chromosomes within parental gametes of egg and sperm. Therefore, with few exceptions, humans like other mammals are born either female or male, and with ovaries and testes, respectively, that produce the sex-specific gametes and steroid hormones that prime the brain for sexual function (i.e., desire, arousal, sexual behavior, and pleasure). However, from a sociological perspective, the binary conception of men and women as somehow biologically fixed extensions of male and female genetic or chromosomal sex has been challenged. In particular, the socially constructed concept of “gender” has replaced “sex” with a fluid spectrum that includes culturally defined expressions of masculinity and femininity, as well as both together, trans, or neither (see Haig, 2000, 2004). In the past, scholars referred to “sex roles” in humans and studied sex-role stereotypy within cultures (e.g., Maccoby & Jacklin, 1974). The concept of gender has largely replaced sex in this regard, such that we now refer to “gender roles” and “gendered behavior.” This conflation of sex and gender has led some to claim that the two are both socially constructed and/or so intertwined as to be indistinguishable (e.g., Butler, 1999; Fausto-Sterling, 2019). This argument has become politicized to the extent that some medical schools and their faculty face criticism on social media, and even punitive actions from academic administrators, for teaching sex differences in response to medical treatments, or for using terms like “breastfeeding” instead of “chestfeeding” in a lecture on postpartum mammary gland function (Meyer-Bahlburg, 2023).

The conflation of sex and gender has also crept into a third dimension of people’s lives: their sexual orientations. As an extension of the criticism offered by Foucault (1978) against the essentialist view that heterosexuality is a default, preferred, or even “normal” expression of sexuality (making deviations from it therefore “abnormal”), queer theory (e.g., Butler, 2020; Chandler & Munday, 2011; Green, 2007) now intersects sexuality and gender to examine and criticize how culture, society, and individuals perpetuate gender and sex-based binaries. People are now identified on the basis of being “cisgendered” “transgendered,” “androphilic” and/or “gynephilic” without the need to use “old-fashioned” terms like “straight” or “gay/lesbian” or even “bi”. This is perhaps an advance, but it is also a conundrum. For example, a man who has sex exclusively with men is considered homosexual in his orientation (something also considered relatively immutable) unless he transitions to female, whereupon she is now heterosexual. Nowhere is the three-way conflation of sex, gender, and orientation more obvious than the current moniker used to describe all sexual minorities as “LGBTQIA2+.” Fluidity and flexibility are now assumed for gender, sex, and sexuality, creating intersecting spectra for individuals to define themselves over the lifespan, and especially with reference to, and differentiation of, their partnered versus solitary sexual desires, behaviors, and self-defined sexuality. Although van Anders’ (2015) sexual configurations theory provides an important and insightful framework to conceptualize and integrate these phenomena, the integration can be problematic when sex and gender are conflated to mean the same thing.

Certainly, questioning the binary nature of sex and/or gender is a valid scientific exercise, yet also an uphill battle. As noted above, there are naturally occurring variations in embryonic development following fertilization, such as androgen insensitivity syndrome (AIS) in which genetic males can appear by external genital anatomy as female at birth (due to the lack of androgen-facilitated sexual differentiation); and congenital adrenal hyperplasia (CAH) in which genetic females are androgenized in utero via adrenal androgen release and appear as fully or partially differentiated males at birth. Note that in both cases it is the external genital structures that are typically used to determine the natal sex. Chromosomal anomalies also occur, called aneuploidies, where there are missing or extra copies of chromosomes that lead to a deviation from sex chromosome pairs other than XX and XY. However, gonadal status is still a highly consistent and defining binary characteristic of sex; and more crucially, none of the variations are considered a “new sex” or a “degree” of male or female sex, nor do they change how humans reproduce (see Findley et al., 2005). They also do not necessarily lead the individuals to a gender identity that is inconsistent with their genetic sex. However, when considering their sexual orientation, studies conducted with individuals with disorders of sex development have found that 26% of severe and mild salt-wasting CAH women had bisexual or homosexual sexual fantasies, whereas those who had non-heterosexual partners did not differentiate significantly from the control group (Zucker et al., 1996). Moreover, 46, XY women with complete AIS almost always identify as heterosexual (Hines et al., 2003), whereas a consistent cross-cultural pattern is observed in girls with 5α-reductase deficiency syndrome. These individuals are XY males who lack normal male-typical external genital organs at birth due to attenuated fetal DHT synthesis from testosterone. However, increased testosterone secretion during puberty often masculinizes body stature and their external genital organs, leading such individuals to switch to a self-identified male sex and gender identity (Al-Attia, 1996; Sobel & Imperato-McGinley, 2004).

The real issue lies in how we define ourselves as sexual individuals. This is where conflation of sex- and gender-related phenomena often occurs. These phenomena can be conceived of as separate (as proposed by Money, 1987), although conscious awareness of our sexuality sums them in various ways:

1. We have a natal sex (typically “assigned” legally on a birth record following observation of a newborn’s genital anatomy);
2. We have a self-recognition of that sex that comes into focus during early childhood and is reinforced by one’s physical (usually genital) anatomy;
3. We have an assumed and/or self-identified gender. This can be “cis” if congruent with one’s sex, “trans” if opposite to one’s sex, or somewhere on a spectrum between the two that either encompasses both sexes on one end (as in “two-spirited”), or neither on the other end (as in “agendered”);
4. As we progress through adolescence and adulthood, we have a spectrum of desire for sexual and/or romantic, intimate interactions with others (i.e., allosexual), or lack thereof (as in asexual or aromantic); and
5. We have a particular sexual attraction and/or romantic orientation that is expressed as arousal toward and preference for persons of one sex or the other (as in heterosexual, homosexual), persons with features of one gender or the other (as in androphilic or gynephilic), for persons of both sexes (as in “bisexual”), persons with no particular sex or gender expression (as in “pansexual”) or that is defined more by romantic attraction than sexual attraction to a person (as in “asexual”). This can also include paraphilic interest in specific animate or inanimate stimuli or behaviors outside of what cultures consider “normal” (King, 1945), but that induce sexual arousal and have been associated with sexual pleasure (Pfaus et al., 2012, 2020; Quintana et al., 2022).

This issue of relatedness between these phenomena relies on three different, but interactive, levels of analysis: (1) the DNA/chromosomal level and its implications; (2) the anatomical/physiological level and its implications; and (3) the sociocultural/environmental level and its implications. On the one hand, a cascade of events and processes determined by different genes and other factors dictate who or what we are as sexually reproducing individuals (true of all sexually reproducing species). On the other hand, whether a person identifies as a cis- or transgendered female or male with a sex-typical anatomical or physiological phenotype who engages in sex-typical or atypical behavior, is a function of psychology, sociology, environment, the chosen degree of hormonal/surgical intervention, and the epigenetics of sexual and romantic pleasure, displeasure, or even aversion, that is experienced during sexual and romantic intimacy. Sitting squarely in the middle of these phenomena, being both biologically determined and experientially dependent, is the brain.

## What Is Sex?

For as simple or straightforward as it used to seem, sex is an incredibly complex concept. It can be defined broadly as a set of biological attributes in animals (CIHR, 2018) or simply as a system that defines and distinguishes males from females in all species that reproduce sexually (Joel & Fine, 2022; Purves et al., 2000). Sex is largely and perhaps almost exclusively defined in terms of DNA and chromosomal structure, but is also influenced by hormonal makeup, and typically determined by the observable anatomy and physiology of external genital structures (e.g., clitoris, penis, vagina, etc.) that have been sexually differentiated. Sexual dimorphisms also exist in certain structures and systems of the brain, spinal cord, and in brain function, endocrine function, cognition, and behavior (e.g., Hyde, 2014; Miller & Halpern, 2014). The classic process of sexual differentiation (McCarthy & Arnold, 2011; Phoenix et al., 1959) occurs in two phases: an organizational phase during a critical period of perinatal development in which the genitals and internal gonads and central nervous system differentiate into a male anatomical and physiological phenotype versus a female “default”; and an activational phase during puberty in which the differentiated structures are now stimulated by gonadal steroid hormones to activate secondary sex characteristics, anatomical, physiological, and neural substrates of sexual behavior, and to strengthen already-present cognitive sex differences and brain functions. However, this model has been updated to a more complex interplay of several genes and their different levels of expression, hormones, the environment, and critical periods in the phenomenon of sexual differentiation (McCarthy & Arnold, 2011; Rey et al., 2020). There is still a linear relationship in the sexual differentiation of most individuals that leads from genetic/chromosomal sex to anatomical/physiological sex. Most people think that this extends to sexually dimorphic brain function and gendered behavior. Although that can be true, it is not the case for everyone. In fact, it has been suggested on the basis of functional anatomy that the brain is a mosaic of “female” and “male” phenotypes in most individuals (Joel, 2011, 2021; Joel et al., 2015; Rippon et al., 2021), though the relationship of that to gendered behavior is tenuous at best.

## DNA and Chromosomal Structure

The proportion of human individuals that have a genetic makeup composed of female XX or male XY sex chromosomes is estimated above 99% (e.g., Blackless et al., 2000). The small proportion of intersex individuals or those with disorders of sex development (DSDs), represent a congenital condition in which a person’s chromosomal, gonadal, and/or phenotypic sexual characteristics are atypical (Lee et al., 2006), and do not fit male- or female-typical medical and social standards, or simply where the chromosomal sex is inconsistent with phenotypic sex (e.g., Allen, 2009; Sax, 2002). Among the several DSDs, an individual may lack or have an extra sex chromosome that leads to inherited hormonal conditions that impact sexual development and differentiation (Eggers et al., 2016). Depending on the underlying condition, DSD individuals can be as healthy as any other (ISNA, 2008), yet some DSDs are associated with atypical development of anatomical sex, excess or deficiency of steroid hormones, infertility, and a higher risk of neonatal death in rare cases, among other conditions (Lee et al., 2006; Witchel, 2018).

DSDs can be classified into several categories, including chromosomal, gonadal, and anatomical abnormalities (see Audí, 2011; Witchel, 2018), where CAH and mixed gonadal dysgenesis are among the most common causes of ambiguous genitalia in newborns (Thyen et al., 2006), and can be potentially life threatening in some cases (Allen, 2009). When it comes to reproduction, and depending on the condition, DSD individuals can create either eggs or sperm. There are no “intersex/DSD sex chromosomes” that would make this different. This is also true even in extremely rare DSD individuals who develop a partial testis and ovary, with varying capabilities of producing both sperm and eggs (Irmak, 2010). There have been cases of some of these individuals who have been self-fertilized and led a pregnancy to term, where the offspring inherited only a pair of sex chromosomes, XX or XY (Minowada et al., 1984; Schoenhaus et al., 2008; Williamson et al., 1981). Therefore, genetic sex matters in many medical and psychological conditions that affect sexes differently, and it can make the difference to an effective treatment (see Cahill, 2006; Duchesne et al., 2020; Institute of Medicine, 2001; Regitz-Zagrosek, 2012). However, not all sexual traits are encoded in our sex chromosomes.

## Sexually Differentiated Anatomy and Physiology

A new embryo leading to a male or female individual is determined genetically, yet the phenotypical manifestation does not always follow the genetic structure. For instance, disorders of sex development are not genetically predisposed but congenital, and individuals can get copies of either sex chromosome, or a hormonal makeup that leads to a degree of ambiguous or not-fully developed reproductive organs, among other differences leading to a plethora of variability in phenotypic sex characteristics (Kim & Kim, 2012). Moreover, in the sexual differentiation process in utero, the Y sex chromosome carries the *Sry* gene, which activates the testis-forming pathway in an embryo whose gonads start out bipotential, capable of differentiating into either testes or, without androgenization and the activation of Anti-Müllerian hormone, ovaries. This gene modulates the *SOX9* gene that triggers the development of testes. Thus, high levels of *SOX9* protein are needed for the programmed development of testis (de Santa Barbara et al., 1998), otherwise a DSD would be expected (although it has been proposed that sex chromosomes establish sexually dimorphic interactions with the autosomes long before gonadogenesis; Engel, 2018). Interestingly, the *SOX9* gene was found to be regulated by “enhancers” present in what geneticists call “junk DNA” that increase or decrease *SOX9* gene activity. Thus, XX subjects with more enhancers developed testes, whereas XY subjects with fewer enhancers developed ovaries (Croft et al., 2018), contrary to what is expected if we only consider sex by what is inherited through sex chromosomes. There are also other factors that are not present in our sex chromosomes that modulate sexual differentiation. For instance, *WNT4* is a gene located in Chromosome 1 that promotes the sex development of females through cell proliferation of early gonads (Chassot et al., 2012), whereas its absence is required for the sexual development of males (Jameson et al., 2012). DSDs arising from AIS and CAH can also lead to partial or full gonadal development that is at odds with an individual’s chromosomal structure due to demasculinization of genetic males in AIS or defeminization of genetic females in CAH.

## Sexually Differentiated Brain and Behavior

Sexual differences can be found in brain anatomy, morphology, connectivity, function, and in different behaviors and cognitive abilities. The dividing line among different types of sex differences is rather blurry. McCarthy et al. (2012) proposed three categories to organize and operationalize them: Type I, morphological, physiological, or behavioral differences that are present exclusively or predominantly in either males or females (e.g., copulatory behaviors, courtship displays, etc.); Type II, differences that exists in a continuum where males and females may fall at any point, yet the group averages are sex-specific (e.g., pain thresholds, food preferences, fear, etc.); and Type III, instances where males and females tend to converge, yet they diverge in response to challenges (e.g., parental behavior, problem—solving strategies, responses to stress, etc.). For instance, genetic studies have found that sex chromosomes have a direct effect of sex differences in brain and behavior (Ngun et al., 2011). Regional and global network connectivity analyses of a sample of 949 people between 8 and 22 years old, showed that males had greater within-hemispheric connectivity and enhanced modularity and transitivity, whereas females predominated in between-hemispheric connectivity and cross-module participation (Ingalhalikar et al., 2014). Additionally, structural and functional sex differences in the hypothalamus have been reported (Swaab et al., 2001) which appear to vary across the life span (Swaab et al., 2003), as well as the menstrual cycle (Protopopescu et al., 2008). Similar findings have recently been replicated through brain imaging with a large adult sample (i.e., > 5000 males and females), where males had higher raw volumes, raw surface areas, and white matter fractional anisotropy, whereas females had higher raw cortical thickness and higher white matter tract complexity, among other findings (Ritchie et al., 2018). Postmortem studies have also shown sexually dimorphic differences in humans. For instance, the central area of the bed nucleus of the stria terminalis, known to be sexually differentiated and involved in the orchestration of sexual behavior in rats (e.g., Emery & Sachs, 1976), is larger in men than in women (Allen & Gorski, 1990). Interestingly, Zhou et al. (1995) showed that the size of this brain area in male-to-female (MtF) transgender individuals was similar to that found in cisgendered women. Similarly, Taziaux et al. (2016) studied kisspeptin expression using postmortem tissue from the arcuate nucleus of the hypothalamus, which is robustly larger in women than in men (Hrabovszky et al., 2010). They found the female-dominant sex difference in this neuronal population, but also that the kisspeptin expression in MtF trangender individuals was also similar to that found in cisgendered women, a finding that could reflect either preexisting distributions or ones that changed following MtF transgender hormonal manipulations. Animal studies have shown sex differences are also present in the distribution of different neuropeptides (e.g., Clarkson & Herbison, 2006; de Vries & Panzica, 2006) and steroid hormone receptors (e.g., Kühnemann et al., 1994; Lu et al., 1998; Quadros et al., 2008), showing the extent to which genetic sex accounts for male–female differences in the brain. However, it is important to note that differences in tissue fixation, processing, and analysis between well-prepared samples taken from animals relative to those taken from post-mortem human tissues can alter the magnitude, if not also the directionality, of the results. This is one potent criticism of all post-mortem brain tissue studies in humans (see Lawrence & Zucker, 2014). Whereas the *Sry* gene is not the only factor explaining sex differences, it possesses a direct role on the morphological and functional sex differences found in dopamine neurons in the ventral tegmental area of rats, even prior to exposure to gonadal steroid hormones during in utero development (Reisert & Pilgrim, 1991; for a detailed list of neuroanatomical, chemical, and behavioral sex differences see Tables 1, 2, and 3 in Ngun et al., 2011). Yet, do these differences translate into behavioral differences? This is where things get difficult.

Although humans show a certain degree of sex differences in other aspects such as cognitive function (Hampson, 2002; Miller & Halpern, 2014), reactivity to drugs like analgesics (Mogil, 2020), and susceptibility to mental health problems and neurological disorders like addiction, schizophrenia, and Alzheimer’s disease (Androvičová, et al., 2021; Ferretti et al., 2018; Gillies & McArthur, 2010), they do not display sexually dimorphic behavior outside of sexual and reproductive specializations such as pregnancy, parturition, lactation, and penile ejaculation; with differences in sexual arousal and orgasm being related to differences in sexual acculturation, education, and appropriate and competent stimulation (Pfaus et al., 2016). Humans do not have sexually differentiated sexual behaviors, whereas other mammals display more global patterns of sexually dimorphic copulatory behaviors (e.g., mounts, intromissions, and ejaculations in males; solicitations, pacing behaviors, and lordosis in females; e.g., Pfaus et al., 2003) and parental behaviors (e.g., nursing, pup grooming and retrieval in females, parental behaviors or infanticide in males; Wynne-Edwards & Timonin, 2007). Although the brain has numerous sexual dimorphisms in terms of the size of various cortical, limbic, hypothalamic, and brainstem structures, most of which are larger in females relative to males (e.g., de Vries & Forger, 2015; Sacher et al., 2013), the sexual dimorphism in any of them is hard to relate to specific cognitive or behavioral functions and, as mentioned above, they appear in more of an individual mosaic than an absolute or completely fixed sex-dependent pattern (e.g., Joel, 2011; Rippon et al., 2021). For example, the corpus callosum that connects both hemispheres of the brain is generally larger in females than in males. This may underlie the greater bilaterality of language function in women relative to greater unilaterality in men (Hampson, 2002; Miller & Halpern, 2014). However, this is by no means established causally nor is it known whether it applies to other cognitive sex differences. Likewise, the slight but significant sex differences in cognitive abilities, like female superior fine motor coordination and male superior spatial skills, are not (yet) determined by any particular dimorphic brain structure(s). And beyond obvious primary and secondary sexual characteristics related directly to reproduction, natal females and males possess different anatomical and physiological characteristics related to physical ability and hence to different sports disciplines. These include height, skeletal structure, muscle mass and area, jumping ability, diving ability and capacity, and short distance sprinting, to name a few (see Hilton et al., 2021; Weiss, 2024). In these cases, natal males that have gone through puberty and are competing at the highest levels always have an advantage.

Similar circumstances exist in animals. For example, the medial preoptic area (mPOA) was one of the first regions in which lesions were shown to disrupt or abolish male sexual behavior (Larsson & Heimer, 1964). Within the mPOA there exists a “sexually dimorphic nucleus” (SDN) that is approximately three times larger in males than in females of a variety of mammalian species (Gorski et al., 1978; Raisman & Field, 1971). However, female Japanese macaques form stable female-female sexual relationships during the breeding season in which one mounts the other repeatedly with genital rubbing and pelvic thrusting (Vasey, 2002a; b). At first glance, it seemed likely that these macaques would have a smaller sex difference in their homologous SDN region given that the females display a “masculine” behavior (e.g., mounting). However, the dimorphism of their SDNs was identical to other macaque species that do not display this behavior (Vasey & Pfaus, 2005). Indeed, in female rats, lesions of the mPOA disrupt or abolish sexual solicitations (Hoshina et al., 1994), indicating that this dimorphic structure is specialized for appetitive sexual behaviors in females that are complementary to appetitive sexual behaviors in males (Pfaus et al., 2003). And female rats will mount sexually inactive male rats with pelvic thrusts and dismounts that resemble a complete male intromission pattern (Afonso, et al., 2006). This type of mounting is a super-solicitational behavior that is disrupted or abolished by lesions of the mPOA, among other hypothalamic regions (Afonso et al., 2009). Adult female rats are approximately half to two-thirds the size of males. Females have higher daily activity levels compared to males (Lightfoot, 2008), and when food-restricted, females display relative faster and longer running rates in running wheels compared to males (Jones et al., 1990). Female C57BL6/J wild-type mice have greater maximal exercise capacity for running distance than age-matched males (Oydanich et al., 2019). Even when matched for weight or muscle mass, females still maintain greater exercise capacity than males. This is due in this strain of female mice to increased type I and decreased type II myosin heavy chain fibers in the soleus muscle relative to males, which provides them with increased endurance and higher resistance to fatigue (Haizlip et al., 2015). In that study, the differences were largely due to patterns of circulating testosterone and estradiol. Thus, despite the relative differences in size among male and female rodents, a rodent Olympics of endurance sports would favor females over males, whereas abilities related to height would favor males over females. These and many other, brain or behavioral, sex differences have been found through animal studies. However, not every finding has been replicated in humans (e.g., Fliers et al., 1986). It is important to keep in mind that the lack of replicability can be explained, in part, by methodological factors regarding the techniques used and their limitations (e.g., differences in post-mortem tissue processing and analysis), in addition to the possibility of species’ differences.

Regarding the literature on sexual dimorphism within the human brain, alternative perspectives to the binary such as the monomorphic or mosaic hypothesis has gained traction the past few years. This perspective challenges one of the most consistent and replicated findings in the sexual dimorphism literature: overall size and volume differences between the sexes. As previously mentioned, this consistent replication has been found in neonates and onward, peaking at adolescence and stabilizing in early adulthood, with ranges going from 4–20% depending on what is compared, the age of the sample, study design, etc. (see Eliot et al., 2021; Ruigrok et al., 2014). Proponents of the monomorphic/mosaic hypothesis claim that, rather than predicting one’s sex on the basis of brain structure and function, it is about the opposite (Joel, 2011). Studies supporting of this view have shown that once body size is accounted for through brain size normalization, sex differences become small, unreliable, and insignificant (Eliot et al., 2021). While several sex differences in volume still persist, these appear to account for a very small percentage of the total variability in different brain structures, potentially explained by other non-controlled factors (e.g., Boles, 2005; Marwha et al., 2017; Potvin et al., 2016, 2018; Sanchis-Segura et al., 2020, 2022).

The monomorphic/mosaic hypothesis has not been exempted from criticism. One of its seminal publications (Joel et al., 2015) was criticized as methodologically faulty and its interpretation misleading (del Giudice et al., 2016), or even as unnecessary corroboration of the obvious inability to distinguish sex on the basis of brain differences relative to gonadal differences (Glezerman, 2016). Furthermore, Joel’s mosaic hypothesis relies on its analysis. Using structural MRI, Chekroud et al. (2016) created a model that was able to identify an individual’s sex from their whole-brain mosaic patterns in brain morphology with incredibly high accuracy (i.e., 93% [95% CI = 89.5–94.9]). They still point out that both perspectives, a mosaic and sexually dimorphic brain, are not mutually exclusive, and that there are undeniable sexually dimorphic statistical patterns between sexes, especially when considering a multivariate approach (Rosenblatt, 2016). Recently, Williams et al. (2021, 2022) claimed that many of the studies reviewed in Eliot et al. (2021) have small sample sizes and inadequate adjustment for global brain size, leading to false positives. Using a large sample size (*N* = 40,028), and after correcting for brain and body size and between regional and global brain measures using the covariate method, they found sex differences in total brain volume, total surface area, and other structural measures. They showed that differences in height only account for 39% of the sex differences in total brain volume, while 409/620 brain measures analyzed showed significant differences with larger cerebral volume of males over females (36%) and vice versa (29%).

New levels of complexity in sex differences research have only begun to be unveiled through new advances in more sophisticated analyses and levels of evidence. For instance, a brain imaging study specifically focused solely on cortical morphology while using multivariate pattern analysis distinguished male and female brains with 97% of accuracy (Luo et al., 2019), whereas a machine-learning algorithm trained with a similar statistical method was able to discriminate male and female brains with greater than 93% accuracy (Anderson et al., 2018). Yet, when using cis- and transgender individuals as part of the sample, a machine learning algorithm was able to distinguish people’s connectivity signatures only with a 48–62% accuracy (Clemens et al., 2020). Meanwhile, the connectome of the nematode worm *Caenorhabditis elegans* which has only recently being determined, showed a high level of sex dimorphism between males and hermaphrodites which is highly conserved across mammals, while demonstrating that species- and behavior-specific sexually dimorphic neural circuits also act together with other neuronal populations to orchestrate other functions related to sexual and other behaviors (Emmons, 2018). This shows that sexually dimorphic biological attributes extend well beyond the realm of sex and sex-related functions, making them much harder to be dismissed. Still, new methodological, statistical, and social considerations should be considered when analyzing, interpreting, presenting, and reporting findings in this hotly debated topic (DeCasien et al., 2022; Fitsch, 2021; Sanchis-Segura et al., 2022). Finally, these findings highlight that even small sex differences can impact behavioral functions in crucial ways. de Vries and Forger (2015) clarify that sex differences extend far beyond the brain and gonads to the entire body. As noted above, sex differences can be found in muscles, fat, liver, the immune system, gut, kidneys, bladder, and placenta, thus ultimately affecting brain and behavior. Furthermore, they highlight that brain sex differences may compensate for the fact that the brain develops and functions in different bodies that otherwise would be more similar between sexes. Similarly, by virtue of being exposed to different experiences, male and female brains would perceive a different world, as well as being perceived differently by it. Therefore, the aggregation of all sex differences, for as small as they may be, would exacerbate differences or prevent others in certain cases (Arnold & Lusis, 2012).

## What Is Gender?

If sex is difficult, gender is a quagmire. The World Health Organization (WHO, 2021) reiterates that gender is a socially constructed concept that people use to describe their own feelings and manifestations of femininity and/or masculinity. For example, orthodox Judeo-Christian-Islamic religious traditions, along with European cultural perceptions, have always conflated gender with sex: two sexes meant two genders and their appropriate “feminine” and “masculine” traits that included appearance, clothing, mannerisms, behaviors (especially sexual behavior and sexual orientation), social status, and self-identification. This conflation forms the foundation of the current problem. Girls that displayed more “masculine” traits were considered abnormal. Such “tomboys” were likely to reject the social norm of what it meant to be a “woman” and thereby reject the social controls imposed by religious and cultural institutions, especially regarding sexual and reproductive traditions like marriage, maternity, and motherhood. Some were more likely to engage in homosexual behavior or to adopt a homosexual orientation. Some went further and incorporated masculine traits completely, living as “sexually inverted” men. Research in the mid-20th century established that girls with CAH were far more likely to display culturally masculine traits, and it was assumed that this was because they had been androgenized in utero (Kung et al., 2024; Money & Ehrhardt, 1972). Similar behavioral results were found in female monkeys that had been androgenized during the critical perinatal organizational period (Goy, 1970), displaying male-like traits of rough-and-tumble play and threat postures during play. Likewise, boys that displayed more “feminine” traits were considered abnormal for identical reasons that spanned the rejection of cultural masculinity, display of a homosexual orientation, and the “sexual inversion” in which they adopted feminine traits and perhaps lived as women. As psychiatry rose in social prominence, the abnormality of so-called “sexual inversions” was codified into the diagnosis of paraphilias, then shifted to disturbances of [hetero]sexual orientation. Gender dysphoria was added to denote children or adults that not only felt they are the other sex or gender, but for whom the forced manifestation of traits of their natal or birth sex caused emotional distress. Of course, the pathologized term “ego-dystonic homosexuality” was removed by consensus of the American Psychiatric Association from the list of paraphilias originally in the *Diagnostic and Statistical Manual of Mental Disorders* (DSM-I; APA, 1952), replaced with “sexual orientation disorder” in the DSM-II (1968), and, thanks to political activism on the part gay and lesbian communities, a vote of the general APA membership in 1974 replaced sexual orientation disorder with “ego-dystonic homosexuality” in the DSM-III (1980), before homosexuality was removed altogether in the 1987 revision of the DSM-III.

In contrast to the European view, other cultures around the world did not assume that a binary of male and female required masculine and feminine behavior appropriate to the bearer’s natal sex. For example, the indigenous Cree nations of North America had six genders: “Iskwêhkân” (a woman who acts/lives as a woman), “Napêhkân” (a man who acts/lives as a man), “Iskwêw ka napêwayat” (a woman who dresses as a man), “Napêw iskwêwisêhot” (a man who dresses as a woman), “Înahpîkasoht” (a woman dressed/living/accepted as a man), and “Ayahkwêw” (a man dressed/living/accepted as a woman; 2-Spirited People, 2021). Other indigenous nations and peoples, like the Iroquoi, Hunkpapa Lakota, Sac and Fox Nations, had three genders, female, male, and both. Quoting the Hunkpapa Lakota Chief Tȟatȟáŋka Íyotake (Sitting Bull): “Our elders tell us of people who were gifted among all beings because they carried two spirits, that of male and female, together in one body.” (2-Spirited People, 2021, p. 2). Quoting the 18th Century Spanish Governor of Alta California, Pedro Fages:

I have submitted substantial evidence that those Indian men who, both here and farther inland, are observed in the dress, clothing, and character of women—there being two or three such in each village—pass as sodomites by profession... yet they are called ‘joyas’ and are held in great esteem. (Fages & Priestley, 1937, p. 33).

Although referred to as “sodomites” (homosexuals) by both the French and Spanish, the French imposed the term “berdache” to an indigenous American person who assumed the dress, social status, and role of the opposite sex (de Vries, 2024). The term in Europe referred to male crossdressers or the more “feminine” partner in a male homosexual dyad. But, these individuals comprised third and fourth genders in addition to natal females and males that acted feminine and masculine, respectively. Thus, although the body may be sexed, the gendered spirit or person residing in that body did not have to conform to the biologically determined sex of that body in terms of traits or behavior.

## Diversity Is the Default

It is difficult to discuss any topic related to sex or gender where, one way or another, “normality” is assumed or tacitly imposed. Whereas a vast proportion of individuals whose self-identified gender conforms to their genetic sex may hint what might be considered a “default” condition in all sexually reproducing species, it should not be confused with normality. Not only this construct is highly nonspecific, but “natural” and “normal” are not synonyms. As pointed out by Sax (2002), an animal giving birth is considered a natural event, yet if the newborn has two heads, the process of genetic recombination and even gestation would likely be considered abnormal.

Although sex is binary at the genetic level, there are several “layers” beyond this that ultimately determine all sexual and gender characteristics of an individual: gonadal phenotype, genital anatomy and physiology, hormones, sexual maturation, gender identity and socialization, sexual orientation, and experiences that ultimately make us the whole sexual beings we are (e.g., Money, 1987). While these factors derive one way or another from our genes, they are also influenced by environmental factors (e.g., radiation, medication, infections, immune actions, maternal hormone levels, etc.), that can alter the process of sexual differentiation (Toppari & Skakkebæk, 1998), along with postnatal experiences that, at an epigenetic level, can alter brain function and cognition. For instance, sexual orientation is one of the most pronounced sex difference traits among humans and animal models used in mate choice (Adkins-Regan, 1988; Ngun & Vilain, 2014). Whereas sexual orientation has a strong genetic component (Bailey & Pillard, 1991; Bailey et al., 2000), and therefore the proportion of people who are heterosexual is overwhelmingly vast, it is also known to be influenced by the environment (Ngun & Vilain, 2014; Patterson, 2008), and is more flexible than previously thought (Carrillo & Hoffman, 2017).

Is there an archetypical “male” or “female”? From a genetic and chromosomal level of analysis, yes. But not from a whole-body level of analysis. No human being fits into a hypothetical sex-based physical archetype as a whole. Second, DSD people exist. Although sexual reproduction requires eggs and sperm, the sexual organization and activation of DSD persons is a living testament to a wider expression as to how female and male physiological phenotypes can present themselves. If sex is a system of characteristics that makes us male or female, why does that have to be reduced to only the genetic level of analysis? Third, beyond the chromosomal level, there are a great deal of variables and sexual characteristics between males and females that cannot be categorized as binary as mentioned above (Fausto-Sterling, 2012, 2019). Fourth, sexual differentiation leads to the development of either male or female genitalia from the very same cells (Hake & O’Connor, 2008). Whereas there are clearly two distinguishable genetic possibilities along with DSD conditions, all other sexual attributes (anatomical or physiological) and any degree of their expressions fall in a range of possibilities. Furthermore, whereas giving an egg or sperm gamete is an unequivocal and exclusive sexually reproducing domain of females or males, respectively, some individuals with DSD conditions can produce both. Should they be considered male, female, both, or an exception? What it is true is that they exist, and, despite being infrequent, they challenge the strict concept of sex as binary beyond the sex chromosomal level.

It is clear that gender as a concept is far more diverse than sex. Because most people are cisgendered, their gender and natal sex are the same and their orientation as androphilic and/or gynophilic simply makes them straight, bi, or gay/lesbian. For trans persons, these definitions become more difficult, and all the more so in the case of people who feel trans as kids and then de-transition as teens or young adults (see Steensma et al., 2011). In addition, how gendered are masculinities or femininities when the norms that define them change within a culture? Is gender still a viable criterion for social status? Perhaps in orthodox and traditional subcultures, but does—or should—a pregnant female-to-male (FtM) trans androphilic person with a beard really raise eyebrows anymore? We offer the following two figures to show how the binary conception of sex, its spectrum of male-like and female-like traits and DSD possibilities, as well as the gender spectrum perspective, can co-exist in models of human sexuality. Although, we do reiterate that sex is not socially constructed, but biologically determined, and binary at the chromosomal level.

**Fig. 1:**
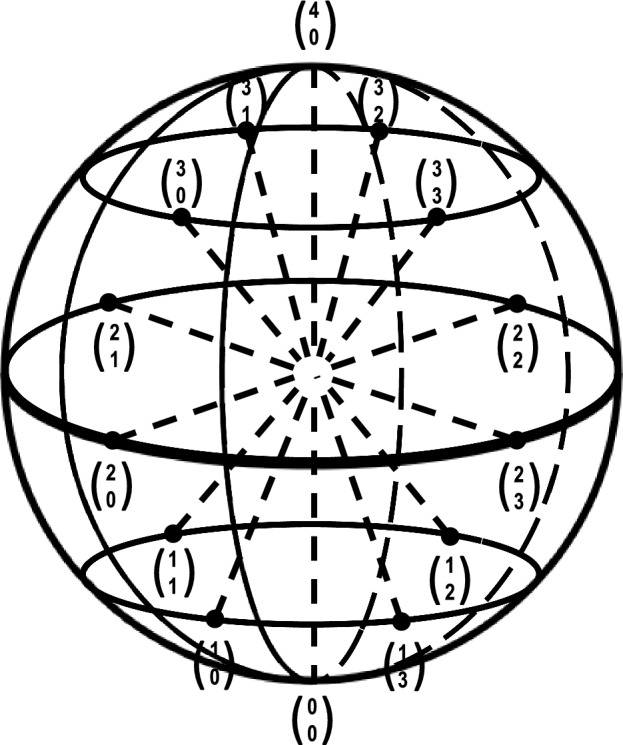
A multidimensional Eshkol-Wachman sphere consisting of orthogonal dimensions representing dichotomous attributes such as sex, gender, sexual orientation, romantic orientation, etc. that intersect to define a person’s sexuality at any one point in time during the lifespan (after Jacobs et al., 1988)

Figure 1 shows an Eshkol-Wachman sphere used originally to define limb segments for movement notation (Jacobs et al., 1988). The sphere has several orthogonal lines corresponding to limb positions that bisect one another at a point in the center. For our purposes, this sphere now represents a person’s sexuality, and the lines represent different elements of sex, gender, and orientation. This three-dimensional set of coordinates define a person along these variables at any moment of that person’s life. Some lines, like sex, may well be stationary for some individuals, whereas other lines, like orientation defined on a Kinsey scale, might be more fluid. The grid defines a snapshot for one person at one particular time during the lifespan. It does not predict how a person’s experience and life history may change the self-definition of sexuality, only that the points upon the grid can change, some more than others.

**Fig. 2:**
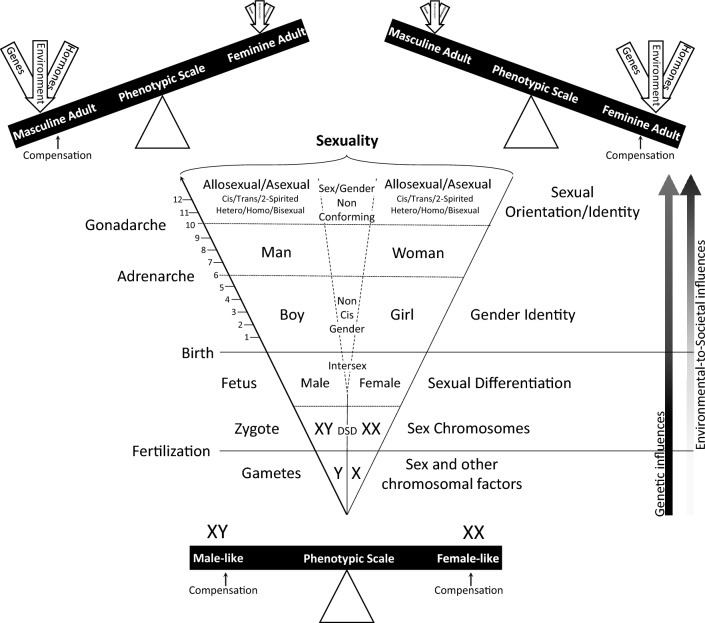
Genetic, physiological, and environmental processes involved in the development of a person’s sex characteristics and gender identity. Post-fertilization, chromosomal factors and transcription stages guide the embryo to develop male or female traits through genes, hormones, and environmental influences, leading to changes in the gonads and genitalia, whereas medical interventions can influence this process for those with DSD. Going into puberty, adrenal androgens increase, initiating adrenarche and other physical changes. Gonadarche follows, marking puberty and enabling reproduction. Sex-specific factors may mitigate undesirable behavioral differences. Gender identity and sexual orientation evolve during adolescence, influenced by genetics, environment, socialization, and experiences. Genetic, hormonal, and environmental influences vary in magnitude among them, as well as across the lifespan and with sexual experience, to determine the degree to which sex differentiation and compensatory processes shape an individual’s sexual characteristics, gender, sexual orientation, and thus their sexuality as a whole. Note: dotted lines represent the blur delimitations between categories and processes. DSD: disorders of sex development; XX or XY: sex chromosome pairs.

Figure 2 shows a summary scheme representing the developmental processes contributing to a person’s sex characteristics and gender identity. Following fertilization, and depending on the chromosomal—pair, more frequently—constitution and other chromosomal factors, a series of sequential stages controlled by a multitude of transcription factors lead a sexually indifferent embryo to acquire male or female characteristics (see Croft et al., 2018; Rey et al., 2020). Thus, under the influence of genes, hormones, and environmental factors, these processes lead to structural and functional changes in the gonads, genital tract, and external genitalia, determining sexually dimorphic anatomical and physiological characteristics. However, for individuals with DSD, the sexual differentiation process would be determined mainly by their condition, with a phenotype that may be influenced by medical interventions after birth. Subsequently, societal gender roles and stereotypes pressure individuals to conform to the gender associated with their sex. Roughly two years before puberty, and without any particular triggering event, there is an increase in adrenal androgens including dehydroepiandrosterone, dehydroepiandrosterone sulfate, and androstenedione, but without increased cortisol levels in both girls and boys (e.g., Quinn et al., 2018). This phenomenon is called adrenarche, which also begins to promote physical changes including the growth of pubic and axillary hair, axillary odor, and acne (Loomba-Albrecht & Styne, 2019). Gonadarche marks the onset of puberty, where gonadal changes grow in size and include the release of gonadal steroid hormones (Levesque, 2011). This is the final maturation process for genital and secondary sex characteristics, the biological milestone in an individual to become capable of reproducing (Witchel & Topaloglu, 2019). A person’s definition of gender grows along the same early lifespan trajectory into adolescence, often reaffirmed through the discovery of their sexual orientation. These maturational processes ultimately crystalize through experiences, setting a person’s sexuality as a whole, which can be relatively more fixed for some than others. It has also been suggested that there are several sex-specific factors caused by sex chromosomal expression or by gonadal hormone actions that compensate for the function of these processes, aiming to prevent or reduce undesirable sex differences in overt functions and behavior, rather than inducing them (de Vries, 2004), which can be enhanced or suppressed over time through socialization processes and sexual experiences (McCarthy & Arnold, 2011). It is understood that this whole process is exclusively and unequivocally genetically driven until the point of fertilization. Meanwhile, environmental influences are susceptible to modulate the genetic expression from that point on, whereas societal influences begin soon after birth, becoming an even stronger modulator of gender identity and sexual orientation over time.

It is important here to consider how a brain that is both biologically determined and experientially dependent integrates sex, gender, and sexual orientation into a relatively immutable sense of one’s own sexuality. We argue that it is through conditioning, both Pavlovian and instrumental, and reinforced both internally and externally by concordant self-schemas that leads to a confidence in one’s own self-definition (Money, 1987; Pfaus et al., 2012). New experiences can change that, and certainly the degree of hormonal and surgical reassignment of one’s genitalia and secondary sex characteristics can also become points of no return for trans persons in their bodily schemas. And trans persons may transition partially or fully, rendering different types of trans persons. Likewise, some cisgendered persons can appear as varying degrees of androgyne. Was David Bowie more or less beautiful as Ziggy Stardust relative to his persona as a pop star a decade later? Was he a Napêw iskwêwisêhot during one phase of his life and then a Napêhkân a decade later? And does any of that change the compelling beauty of his music?

Diversity in sex, gender, and sexual orientation allows for myriad expressions and methods of problem-solving in those domains. Do other animals such as rats have a concept of gender or orientation? Probably not, at least not as humans define it. They do understand sex, sexual arousal, sexual pleasure, and the way that pleasure conditions the desire for certain features of a partner (Pfaus et al., 2012; Quintana et al., 2022). Diversity of sexual preferences, mate choice, and reproductive styles can be observed in a variety of species (Bagemihl, 1999). Diversity in human sex, gender, and sexual orientation includes the fact that lesbian women can choose to have their eggs fertilized in vivo or in vitro by gay men’s sperm, and that they will give birth to offspring who are gynephilic and/or androphilic and who are cis- or transgendered. Of course, the same is true for heterosexual and non-binary individuals, as it is also true that individuals can choose to forgo reproduction. None of that alters their sex or gender.

## Gender/Sex

Other authors instead use the conflated term “gender/sex” (or sex/gender) to highlight how these constructs are intertwined. This new perspective addresses how gender disparities may be reflected in study designs, analyses and interpretations (Hausmann, 2021; Rippon et al., 2014; Schmitz & Höppner, 2014). Rippon et al. argue four points in favor of this perspective: overlap, mosaicism, contingency, and entanglement. “Overlap” refers to the sex similarities between female and male brains. For example, Leonard et al. (2008) claim that gender/sex differences in brain structure and function often disappear when volume is controlled for, though these authors acknowledge that some sex differences do persist. As noted above, “Mosaicism” refers to that brains are not uniformly male or female, but a mixture of both (Joel, 2011), where within the same individual some brain areas may represent a male-like brain and others a female-like brain, leading to erroneous assumptions and interpretations (Joel, 2012). “Contingency” refers to a feedback loop in which environmental influences lead to self-fulfilling prophecies in terms of sex differences observed, for example, as different cognitive performance and social skills. When controlling for confounding factors, previously reported advantages of one gender over the other appear to diminish (e.g., Moè, 2009). Thus, it is hypothesized that male and female neural differences are the product of these intersectional realities and the circle that perpetuates them. Finally, “Entanglement” refers to the malleability of reported sex differences by other manipulations and contexts, highlighting the dynamic, plastic, and interactive nature of brain development (even in adulthood), thus claiming that sex and gender must therefore be “entangled”. Although there is political support for these claims (see Eliot, 2019; Fine, 2021), many are not supported by empirical research, and therefore await more data and replication, especially when an argument as pivotal as the mosaic brain depends largely on the level and type of analyses used (Chekroud et al., 2016; Rosenblatt, 2016).

Another dimension to the conflation of gender and sex is the social constructionist argument. For example, Hyde et al. (2019) argue that ambiguous genitalia are labelled penis or clitoris depending on what cultural expectation of size would be for either (Fausto-Sterling, 2000; Kessler, 1998). Furthermore, they exemplify the use of gender labels given to the sex chromosomes to be contrasted with the fact that, for instance, the X chromosome also plays a role in spermatogenesis (Richardson, 2013). They also argue epigenetic influences dictated by gender stereotypes would lead males and females to be either exposed to or prevented from engaging in male- or female-typical play or work (Clough, 2011). Some of these cultural influences seem arbitrary, like the association of male and masculine with the color blue or female and feminine with the color pink, or are downright incorrect from both neurobiological and neuroendocrinological standpoints, such as the notion of testosterone being a “male hormone” and estradiol being a “female hormone.” Epigenetic changes do indeed modulate several aspects of health (Auger, 2016), anatomy (Tachibana, 2016), brain morphology and function (Ratnu et al., 2017), and even sexual behavior (Holley et al., 2018). Whereas epigenetic influences in our genome and its expression may very well be due to the adoption of, or resistance against, gender roles and stereotypes, these are still believed to be separate from influences related to sex. Thus, epigenetic changes may amplify sex differences in some individuals over the lifespan, whereas in others they may counteract those differences (Cortes et al., 2019).

## The Interaction of Sex and Gender and the Rectification of Terms

The interaction of sex and gender resembles pouring ink in water. As soon as the ink touches the water, it begins to color it, slowly and steadily, until the whole mass of water resembles some degree of the ink’s color, making them seem undistinguishable from one another. On one hand, the influences of gender are unavoidable and ubiquitous regardless of their degree and/or variations from one society to another, cultures, ages, periods of history, etc. Yet, its only constant is its relation to sex. Distilling gender identity and expression yields, in one degree or another, something related to femininity and masculinity, which is undeniably related to maleness and femaleness, or sex for short, just as much as distilling colored water would yield ink and water in our previously mentioned metaphor. On the other hand, reproduction and sexual function are fixed across individuals, societies, cultures, and periods of history. Whereas both are undeniably susceptible to environmental and contextual influences, and regardless of a person’s gender or gender expression, reproduction still occurs only through the fertilization between male and female gametes (at least naturally). Which gamete is passed on is determined only by a person’s sex. Similarly, sexually matured individuals also experience sexual desire and arousal, which in turn influences their behavior in order to pursue sexual pleasure and gratification. For reproduction, then, sex and sexual orientation takes precedent. For social expression and identity, gender is superimposed over sex. However, for sexual behavior, it is a mix of the two.

Gender cannot be reducible to the binary of sex, nor can it be replaced by sex when accounting for differences among individuals in any level of analysis and phenomena where postnatal experience influences the results. Non-binary gender identities and expressions are as valid as any other, and they describe aspects of individuals that other variables like sex cannot. Similarly, gender can only go so far to describe and explain certain aspects of our sexuality, its anatomy, and functioning. Sex and/or gender differences are not inheritably bad or wrong, nor do they imply one is better than the other. They simply demonstrate that we may not be 100% similar beyond our obvious reproductive differences, and that categorization—a valid and necessary scientific exercise—may yield different results depending on the variable used. Demonizing or “cancelling” the study of sex and/or gender differences and interactions harms our understanding and treatment of people’s behavior, mental and physical health, conditions and disorders, performance, etc. (see Cahill, 2006; Duchesne et al., 2020; Hines, 2020), while politicizing any scientific field would likely yield the “flavor” of the political trends in style. There are questions that need to be addressed, and whereas we must remain always open to constructive criticism and attentive to our own biases, scientific quests are those of curiosity and value, yet they may not always yield a comfortable outcome (e.g., the ongoing controversy over the concept of autogynephilia; Bailey, 2023; Blanchard, 1989, 2005; Lawrence, 2006; Moser, 2010; Serano & Veale, 2023).

We argue that understanding the interaction of sex and gender does not require a conflation of the two. The conflation deters from reality and constrains our ability to study them as separate entities that might interact. The conflation creates a scientific and political conundrum that is simply not necessary. No one is harmed when sex differences or similarities are studied, and the degree of differentiation—or similarity for that matter—along with learning helps to maintain diversity in brain and neuroendocrine control of sexual and reproductive behavior. The same is true for gender and its myriad expressions that help to maintain the diversity of individuals within social and cultural settings.

Humans have a predilection for highly nuanced self-definitions and a desire to defend those definitions even if they change over time. Part of the current problem stems from special-interest groups trying to decide which definitions are considered correct, leading to much confusion. Rectification of terms can help to reduce the confusion.

Given the data we have considered in this review, we define sex in mammals as a natal biological and genetic process that leads to anatomical and physiological differences in reproductive function, as female (XX), male (XY), with DSDs conditions being subsumed within the dominant reproductive capability. In contrast, gender is a socially-constructed entity that defines modes of expression (e.g., play, dress, appearance, attitude) as feminine, masculine, both (e.g., Two-Spirited), or as the multitude of degrees/types of agendered or genderqueer expressions. People may define their gender as binary (masculine or feminine), or along a continuum between the two. Cis-gendered persons are those whose gendered expression is consistent with their natal sex. Transgendered persons are those whose gender and gendered expression is opposite to their natal sex. Agendered, genderqueer, or genderfluid persons are those whose gender and gender expressions are more self-defined, and do not conform in any predictive way to their natal sex or to strict cis- or transgender binaries. Although a person’s sexual and romantic attraction and orientation can also come into play in the definitions, a transgendered person can be as gay, mostly gay, bi, mostly straight, straight, or asexual, as any cisgendered person. It all depends on the way one’s self-definition of gender, sex, and sexual and/or gender expression accords with both socio-cultural definitions and those of the individual’s preferred subgroup or subculture. Behavior has been purposely left out of the definitions because it represents the common ground for the integration of all these constructs (and other aspects of one’s personality, experiences, etc.). Whereas there are sex-related behaviors in the animal kingdom like copulation, pregnancy, parturition, lactation, or penile ejaculation that are highly specific—though not fully exclusive—to reproduction, these do not constitute a way to accurately distinguish humans in terms of their sex, gender, sexual orientation, or gender expression, especially when considering their interaction. Therefore, there is no right or wrong, and one’s preferred pronouns do not have to be a source of political or social oppression, or a reason for righteous indignation. As with all human experience, some self-definitions change across the lifespan while others do not. And that experience is different for every human being, despite common group and subculture identities.

In this review we have tried to rectify terms in such a way as to disentangle the concepts of sex and gender, to loosen the choke-hold their conflation is having on sexual science and social policy. On the basis of a similar misuse and misapplication of the term “normal” to mean different things (e.g., “average” versus “ordinary”), King (1945) argued that if the same label is used for different concepts, then the “cooperation essential to successful scientific work is lost, not deliberately but by default.” Further to his point, “Rigorous terminology is not an academic luxury; it is a bed-rock necessity of scientific success” (p. 493). The reality of gender transitioning that includes physical surgery to make one’s body congruent with one’s gender does not mean that the person’s definition of gender is set in stone as sex, even if the desire for physical congruence looks like it is. Likewise, the reality of a person who decides later to de-transition should not be taken as evidence that the initial transition was wrong and should therefore be denied to others. People need to have the freedom to define themselves and the knowledge to be able to make choices about their bodies and their sexualities. That knowledge needs to be based on strong empirical science with terminology that is accurate; not on assumptions or propaganda from special interest groups that seek social or political authority.
